# Biodegradable polycarbonates from lignocellulose based 4-pentenoic acid and carbon dioxide

**DOI:** 10.3389/fchem.2023.1202735

**Published:** 2023-05-04

**Authors:** Weiliang Wang, Rui Qu, Hongyi Suo, Yanan Gu, Yusheng Qin

**Affiliations:** College of Chemistry and Chemical Engineering, Yantai University, Yantai, China

**Keywords:** biobased polymer, CO2-based polycarbonate, lignocellulose based, 4-pentenoic acid, biodegradable (co)polymers

## Abstract

The production of biodegradable polycarbonate by copolymerizing CO_2_ with epoxides has emerged as an effective method to utilize CO_2_ in response to growing concerns about CO_2_ emissions and plastic pollution. Previous studies have mainly focused on the preparation of CO_2_-based polycarbonates from petrochemical-derived propylene oxide (PO) or cyclohexene oxide (CHO). However, to reduce dependence on fossil fuels, the development of 100% bio-based polymers has gained attention in polymer synthesis. Herein, we reported the synthesis of glycidyl 4-pentenoate (GPA) from lignocellulose based 4-pentenoic acid (4-PA), which was further copolymerized with CO_2_ using a binary catalyst SalenCoCl/PPNCl to produce bio-based polycarbonates with vinyl side chains and molecular weights up to 17.1 kg/mol. Introducing a third monomer, PO, allows for the synthesis of the GPA/PO/CO_2_ terpolymer, and the glass transition temperature (*T*_*g*_) of the terpolymer can be adjusted from 2°C to 19°C by controlling the molar feeding ratio of GPA to PO from 7:3 to 3:7. Additionally, post-modification of the vinyl side chains enables the production of functional polycarbonates, providing a novel approach to the preparation of bio-based materials with diverse side chains and functions.

## 1 Introduction

Polymeric materials play an important role in human life and are widely used in packaging, automobiles, coatings, fibers, pipes, and many other areas for their low price and excellent performance. However, these polymers are currently mainly produced from non-renewable fossil fuels and non-biodegradable. Most of these plastics end up being buried or burned after they reach the end of their useful life, resulting in severe environmental problems like resource depletion, white pollution, and increased carbon emissions.

In order to eliminate reliance on fossil fuels and minimize carbon emissions and white pollution, biodegradable polymers derived from green and sustainable monomers have gained popularity in both industrial manufacturing and scientific research during the past few decades. One of the most promising routes is preparing biodegradable polycarbonate materials from CO_2_ and epoxides. Although the carbon atom of CO_2_ is in its highest oxidation state and is highly thermally stable, the presence of two electronegative oxygen ions confers a certain electrophilicity to the carbon atom, allowing it to participate in chemical synthesis (Gibson, 1996; Sakakura et al., 2007; Omae, 2012; Aresta et al., 2013; Artz et al., 2018; Yaashikaa et al., 2019; Xian et al., 2021). In 1969, Inoue successfully synthesized polycarbonate by copolymerizing propylene oxide (PO) and CO_2_, and in the following decades, researchers paid much attention to develop highly efficient catalytic systems for CO_2_ copolymerization. Since the successful synthesis of polycarbonate using Inoue’s diethylzinc/active hydrogen-containing compound catalysts, many catalysts including homogeneous and heterogeneous catalysts have been applied for the copolymerization (Coates and Moore, 2004; Qin and Wang, 2010; Lu and Darensbourg, 2012; Darensbourg, 2017; Poland and Darensbourg, 2017; Kozak et al., 2018; Huang et al., 2020; Plajer and Williams, 2022). In terms of activity and selectivity, the current catalysts are significantly better than before. Therefore, the rapid development of catalysts has greatly accelerated the research development and industrialization of CO_2_-based polycarbonate materials.

Although great progress has been made in the synthesis of CO_2_-based polycarbonates, most research has focused on the copolymerization of CO_2_ with petroleum-based epoxides such as PO and cyclohexene oxide (CHO). The low number of polymerizable monomers results in polymers with relatively single structure and properties. Therefore, the synthesis of CO_2_-based polycarbonates with diverse structure from bio-based epoxides offers ideal solutions to both CO_2_ emission reduction and 100% biobased polymer production (Klaus et al., 2010; Wang et al., 2015; Hauenstein et al., 2016; Parrino et al., 2018; Yang et al., 2021). In 2004, Coates firstly used CO_2_ and limonene epoxide (LO) extracted from orange peel to synthesize CO_2_-based polycarbonates using *β*-diimino zinc catalyst, and proposed a new strategy to synthesize CO_2_-based plastics without relying on petroleum route, leading to a promising trend for the synthesis of full bio-based polycarbonates (Byrne et al., 2004). Subsequently, Wang successfully synthesized polycarbonate from furfural, which is derived from corncobs. The polymer exhibits a low glass transition temperature and good thermal stability, and the unique structure can be used to modify the toughness and thermal stability of traditional PPC materials (Hu et al., 2009). In 2014, Zhang synthesized end-group epoxy methyl undecylenate (EMU) by epoxidation of methyl undecylenate derived from castor oil, and synthesized polycarbonates via copolymerization of EMU and CO_2_ catalyzed by Zn-Co double metal cyanide complexes (Zhang et al., 2014). In 2017, Li prepared soybean oil-based epoxy (SOTE) containing end-group epoxy from soybean oil, and used SalenCoCl/PPNCl to catalyze the copolymerization of soybean oil-based epoxy with CO_2_ to synthesize polycarbonate materials (Chang et al., 2017; Cui et al., 2017). Studies mentioned above have significantly improved the structures and properties of CO_2_-based polymer materials and opened a wide variety of opportunities for the development and utilization of CO_2_-based polymers.

**FIGURE 1:**
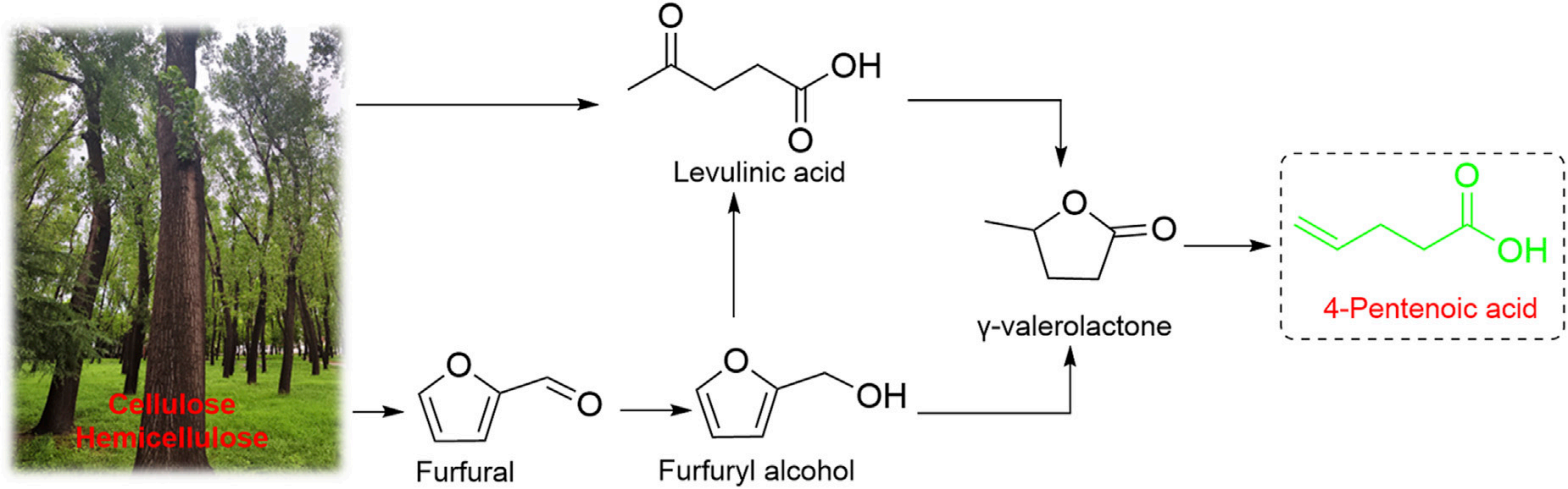
Path ways to produce 4-PA from biomass.

Despite significant progress has been made on full-biobased polymeric materials, the quest for non-food biomass resources with low cost as raw materials remains an important direction for the development of polymer synthesis. Lignocellulose, as one of the most abundant non-food biomass resources on the Earth and is mainly composed of cellulose (30–50 wt%), hemicellulose (20–40 wt%) and lignin (15–25 wt%) (Somerville et al., 2010). The platform compounds produced by lignocellulosic process have large potentials to replace petroleum resources because of their diverse types, lower cost, and better biocompatibility (Isikgor and Becer, 2015; Jing et al., 2019; Lin and Lu, 2021; Wang et al., 2021). One of the most promising lignocellulose-based molecules is *γ*-valerolactone (GVL) (Al-Naji et al., 2019; Raj et al., 2021), which can be synthesized from furfural or levulinic acid (Figure 1). Under SiO_2_/Al_2_O_3_ catalysis, GVLs can be efficiently converted to 4-pentenoic acid (4-PA), which have a unique cheese-like odor and are widely used in fragrances and foods (Al-Naji et al., 2016), as well as a second-generation biofuel and raw materials for monomers of synthetic fibers (e.g., adipic acid, a raw material for the preparation of nylon 66) (Han, 2016; Nobbs et al., 2016; Iglesias et al., 2020). Especially, 4-PA has attracted our attention due to its unique chemical structure. The terminal carboxyl group can be easily modified to produce epoxides, which opens the possibility of producing CO_2_-based polycarbonates original from lignocellulose.

**FIGURE 2:**
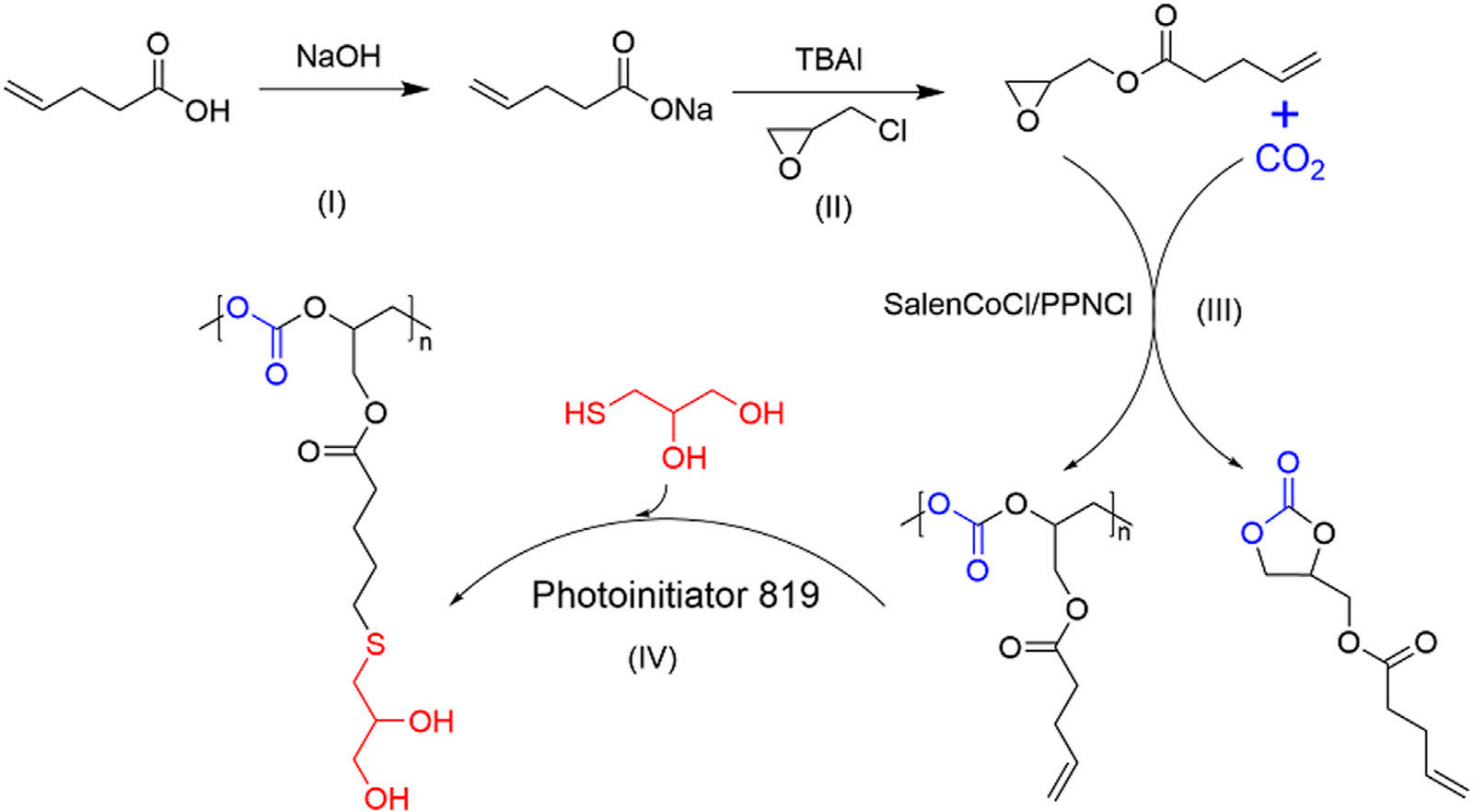
Strategy performed for the preparation of bio-based green polycarbonate.

Herein, we used the reaction of 4-PA with epichlorohydrin to produce the epoxide 4-PA glycidyl ester, and then screened the catalytic system to copolymerize the biobased epoxide with CO_2_ to synthesize full biobased green polycarbonate materials (Figure 2). The vinyl groups in the side chains of the polycarbonates allow for post-modification of the polymers.

## 2 Materials and methods

### 2.1 General information

1-Thioglycerol (98%), epichlorohydrin, photoinitiator 819, sodium hydroxide, tetrabutylammonium iodide (TBAI) were purchased from Macklin Inc. and used without further purification. 4-PA was purchased from Wuhan Lanabai Pharmaceutical & Chemical Co. and purified by reduced pressure distillation. Tetrahydrofuran (THF) was distilled under argon atmosphere from sodium/benzophenone. CO_2_ (99.999%) was used directly as received. (*R*,*R*)-(−)-*N*,*N*-Bis(3,5-ditert-butylsalicylidene)-1,2-cyclohexanediaminocobalt (III) chloride (SalenCoCl) and 5,10,15,20-Tetraphentlporphine aluminum (III) chloride (TPPAlCl) were prepared according to the previous literature (Aida and Inoue, 1983). Bis-(triphenylphosphine)iminium chloride (PPNCl) was purchased from Tianjin Sienna Biochemical Technology Co. All chemicals were reagent grade unless otherwise noted.

Fourier transform infrared (FT-IR) spectroscopy was performed on a Shimadzu IRAffinity-1S FTIR spectrophotometer. ^1^H NMR spectra of the products were performed on a Bruker AV-400 MHz NMR spectrometer, chemical shift values were referenced to TMS as internal standard at 0.0 ppm, the solvent was CDCl_3_ or C_4_D_8_O. The molecular weight (*M*_n_) and polydispersity (*Ɖ*) of the polymer was determined by gel permeation chromatography (GPC) on a Waters 2414 binary system with a refractive index detector, calibrated with polystyrene standards. The column temperature was maintained at 35 °C during the test using THF as the eluent at a flow rate of 1.0 mL/min. Differential scanning calorimetric (DSC) tests were conducted with the heating rate of 10 K/min from 20°C to 180 °C under N_2_ atmosphere. Samples for thermal analyses were all purified. The water contact angle was determined using the JC2000 contact angle measuring instrument.

### 2.2 Synthesis of 4-pentenoate (GPA)

4-PA (50.00 g) was dissolved in acetone (300.00 mL) with stirring at room temperature for 10 min. Then NaOH solution (74.00 g, 30 wt%) was dropwise added with vigorous stirring for 4 h. After filtration, the filtrate was dried at 45°C under vacuum for 24 h to obtain 4-PA sodium salt (4-PANa).

GPA with epoxide groups were obtained through the reaction of the prepared 4-PANa (0.37 mol, 45.00 g) and epichlorohydrin (ECH) (2.14 mol, 198.00 g) at 130°C for 0.5 h. Then, TBAI (2.80 mmol, 1.05 g) was added, and the reaction mixture was stirred for another 1 h. The reaction mixture was further cooled to ambient temperature and centrifuged for 5 min at 6,000 rpm to remove the unreacted 4-PANa from the suspension solution, and then the clean GPA monomers were obtained after excess ECH was removed using a vacuum rotary evaporator and decompression distillation.

### 2.3 Synthesis of polycarbonates from GPA and CO_2_

The synthesis of polycarbonates from GPA (0.02 mol, 3.30 g) and CO_2_ (3 MPa) was performed in a pre-dried 10 mL autoclave in the presence of catalyst SalenCoCl (0.04 mmol, 25.00 mg) and cocatalyst PPNCl (0.04 mmol, 23.0 mg). The autoclave was filled with CO_2_ at a pressure of 3 MPa. The reactions were performed at different temperatures and reaction times with magnetic stirring at a stirring speed of 1,000 rpm. The crude products were dissolved in dichloromethane (DCM, 5.00 mL) and further precipitated through dropwise addition into 50.00 mL ethanol to remove the catalysts, unreacted raw materials, and products with low molecular weight. The purified polycarbonates were obtained after vacuum drying at 25 °C for 48 h.

### 2.4 Synthesis of polycarbonates from GPA, PO and CO_2_

The synthetic procedure for the terpolymerization of the GPA, PO, and CO_2_ was similar to the above copolymerization procedure. The reactions were conducted with three mole ratios of GPA/PO, 3:7, 5:5, and 7:3. Correspondingly, the volumes of the GPA monomers in these three experiments were 0.90 mL (6.40 mmol), 1.50 mL (10.70 mmol) and 2.10 mL (15.00 mmol), and the volumes of the PO were 1.05 mL (14.90 mmol), 0.75 mL (10.70 mmol) and 0.45 mL (6.40 mmol), respectively. After the reaction, the products were dissolved in DCM, precipitated in ethanol, and then dried to obtain the purified polycarbonates.

### 2.5 Post-modification of polycarbonate with 1-mercaptoglycerol

Photoinitiator 819 (8.40 mg, 0.02 mmol), polycarbonate of Table 1, entry 3 (4.18 g), and 1-mercaptoglycerol (2.17 g, 0.02 mol) were dissolved in anhydrous THF, and further irradiated by ultraviolet light for 4 h. After the reaction, the solution was concentrated using a rotary evaporator and then precipitated in cold ethyl ether. After filtration, the filtrate was dried at 45°C under vacuum overnight to obtain the post-modification of polycarbonate.

**TABLE 1:** The copolymerization of GPA with CO_2_ catalyzed by SalenCoCl/PPNCl.

Entry^a^	t (h)	T (°C)	P (MPa)	Selectivity^b^ (%)	Conversion^c^ (%)	TOF^d^ (h^-1^)	*M*_n_ ^e^ (kg/mol)	*Đ* ^e^
1	8	25	3	94	84	53	4.8	1.26
2	12	25	3	92	99	41	15.9	1.60
3	16	25	3	91	99	31	13.3	1.60
4	24	25	3	79	99	21	6.2	1.33
5	48	25	3	78	99	10	6.3	1.35
6	24	0	3	95	45	9	3.6	1.31
7	48	0	3	93	76	8	6.0	1.34
8	8	40	3	23	96	62	/^f^	/^f^
9	16	25	1	56	99	31	10.2	1.55
10	16	25	5	66	99	31	17.1	1.67

^a^ The reaction was performed in a pre-dried 5 mL autoclave, with GPA (0.02 mol, 3 mL), and SalenCoCl catalyst (0.04 mmol, 25.0 mg), PPNCl (0.04 mmol, 23.0 mg).

^b^ Selectivity for polymer over cyclic propylene carbonate.

^c^ GPA, was converted to both poly (carbonate-*co*-ether) and cyclic carbonate and GPA, was not observed in the ^1^H NMR, spectra of entries 2-5 and 8–10.

^d^ The TOF, represents the conversion of GPA, to products including both polymer and cyclic carbonate based on [Co] centers.

^e^ Determined by Gel Permeation Chromatography in THF, at 35 °C calibrated against polystyrene standards.

^f^ no pure polycarbonates were obtained.

## 3 Results and discussion

### 3.1 Synthesis of GPA monomers from 4-PA

GPA was synthesized by the nucleophilic substitution reaction between epichlorohydrin and the sodium salt of 4-PA. 4-PANa was prepared by the saponification reaction between pentenoic acid and aqueous NaOH solution in acetone, and further reacted with epichlorohydrin using the phase transfer catalyst tetrabutylammonium iodide (TBAI) to obtain 4-PA glycidyl ester in 91% yield.

**FIGURE 3:**
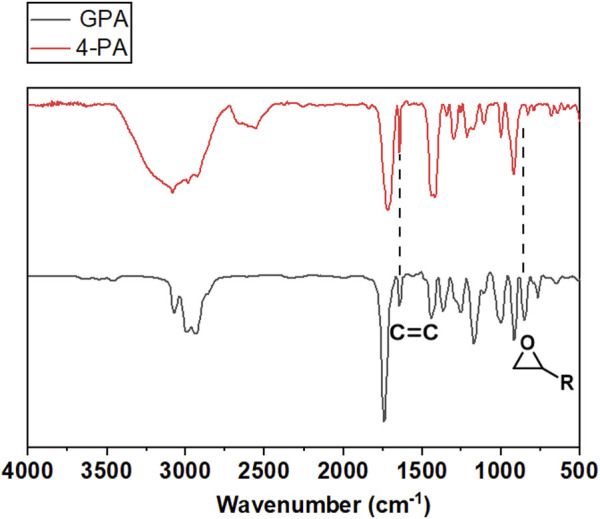
FT-IR spectra of 4-PA and GPA.

**FIGURE 4:**
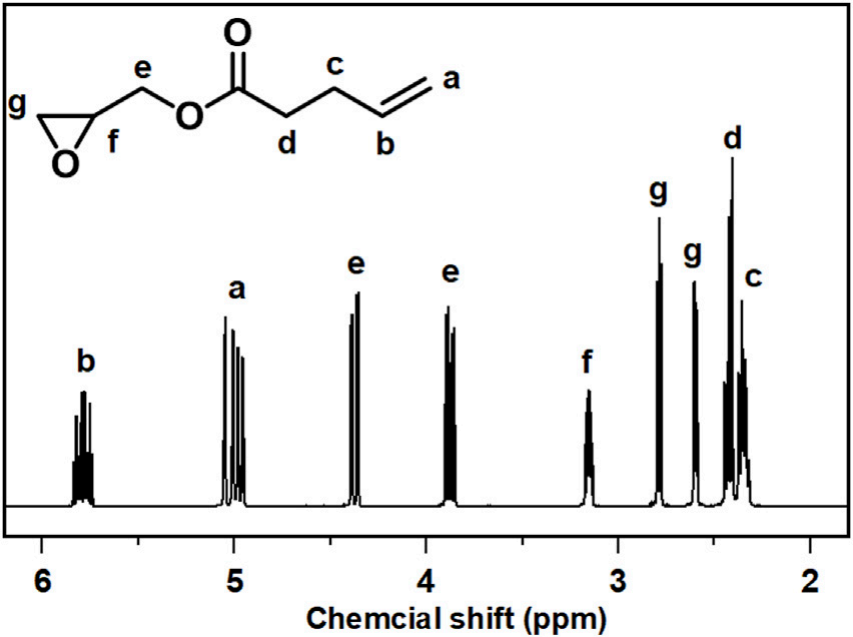
^1^H NMR spectra of GPA (CDCl_3_, 400 MHz).

As depicted in Figure 3, the produced GPA monomers were analyzed using FTIR spectra. Compared to the spectra of 4-PA, the spectra of GPA monomer displayed a new characteristic absorbance peak at 855 cm^−1^, which was attributed to epoxy groups and suggested that the epoxy groups were successfully inserted into the terminal of the fatty acid chains. The disappearance from the typical signals of hydroxyl group (-OH) of -COOH between 2700 and 2500 cm^−1^ indicates that the carboxyl group of 4-PA was properly converted. Identifiable signals at 1,640 cm^−1^ and 3,008 cm^−1^, corresponding to the (CH-CH) and (CH = CH) of vinyl groups on the unsaturated fatty acids chains, were also detected, suggesting that the original vinyl groups of the unsaturated fatty acids chains were maintained. These characteristic chemical structures were also demonstrated by ^1^HNMR spectroscopy. As shown in Figure 4, the characteristic hydrogen atoms of the GPA monomers were as follows: b, 5.7 ppm; a, ∼4.8 ppm; e, ∼4.3 ppm and 3.7 ppm; f, ∼3.1 ppm; g, ∼2.5 ppm and 2.7 ppm; d, ∼2.4 ppm; and c, ∼2.3 ppm. These expected spectral results further confirmed the successful introduction of the oxirane group and demonstrated the interior double bonds retained on the linear fatty acid chains, offering a promising potential site for further synthetic modification.

### 3.2 Synthesis of polycarbonates from GPA monomers and CO_2_

**FIGURE 5:**
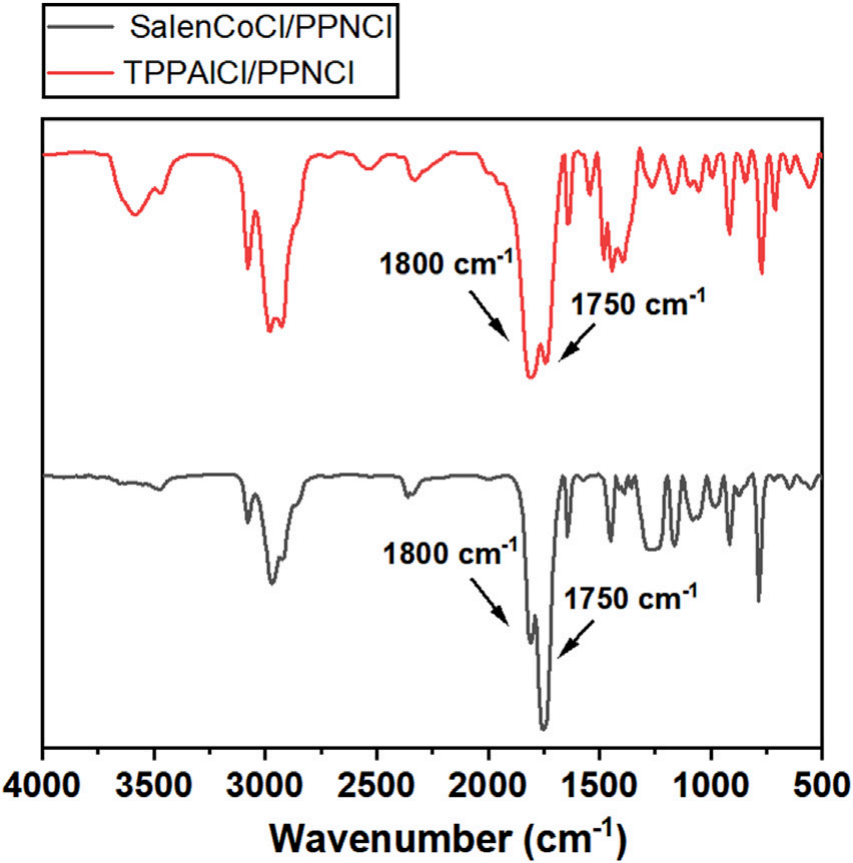
FT-IR (CH_2_Cl_2_) spectra of polycarbonates obtained from the copolymerization of CO_2_ and GPA catalyzed by TPPAlCl/PPNCl and SalenCoCl/PPNCl.

**FIGURE 6:**
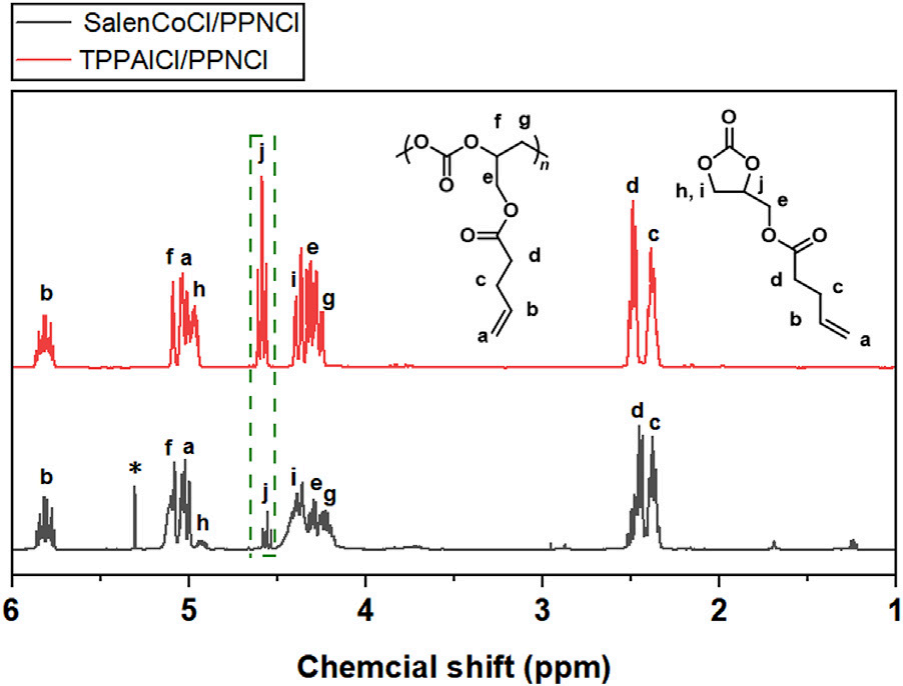
^1^H NMR spectra of polycarbonates obtained from the copolymerization of CO_2_ and GPA catalyzed by TPPAlCl/PPNCl and SalenCoCl/PPNCl (CDCl_3_, 400 MHz).

To achieve high selectivity of polycarbonates, two different catalysts involving TPPAlCl/PPNCl and SalenCoCl/PPNCl were used to catalyze the copolymerization. TPPAlCl/PPNCl is a highly efficient catalytic system for copolymerization of PO with CO_2_ (Jung et al., 1999). The active center of the system is aluminum, which is considered to be green catalyst with no harm to the soil and living creatures (Wang et al., 2018). The catalytic reaction was carried out at 60°C for 24 h using GPA/TPPAlCl/PPNCl with a molar ratio of 500:1:1. FTIR spectra showed characteristic absorption peaks for cyclic carbonate and polycarbonate at 1800 cm^−1^ and 1750 cm^−1^ (Figure 5), respectively, and further analysis by ^1^H NMR spectroscopy revealed up to 35% cyclic by-products (Figure 6).

To improve the polymer selectivity of the copolymerization reaction, another catalytic system SalenCoCl/PPNCl, which proved to be an efficient binary catalyst for CO_2_ copolymerization with high reactivity and selectivity (Lu and Wang, 2004; Cohen et al., 2005; Lu et al., 2006), was used to catalyze the copolymerization of GPA with CO_2_. FTIR spectra showed characteristic absorption peak for cyclic carbonate at 1800 cm^−1^ was still present (Figure 5). Further calculation the conversion of GPA and the polymer selectivity by ^1^H NMR spectrum. The reaction catalyzed by SalenCoCl/PPNCl achieved nearly 100% conversion of GPA within 8 h, demonstrating much higher activity than TPPAlCl/PPNCl. The polymer selectivity of the reaction was calculated to be more than 95%. The absence of ether linkage peak at 3.5 ppm suggests that no polyether was produced during the reaction (Figure 6).

**FIGURE 7:**
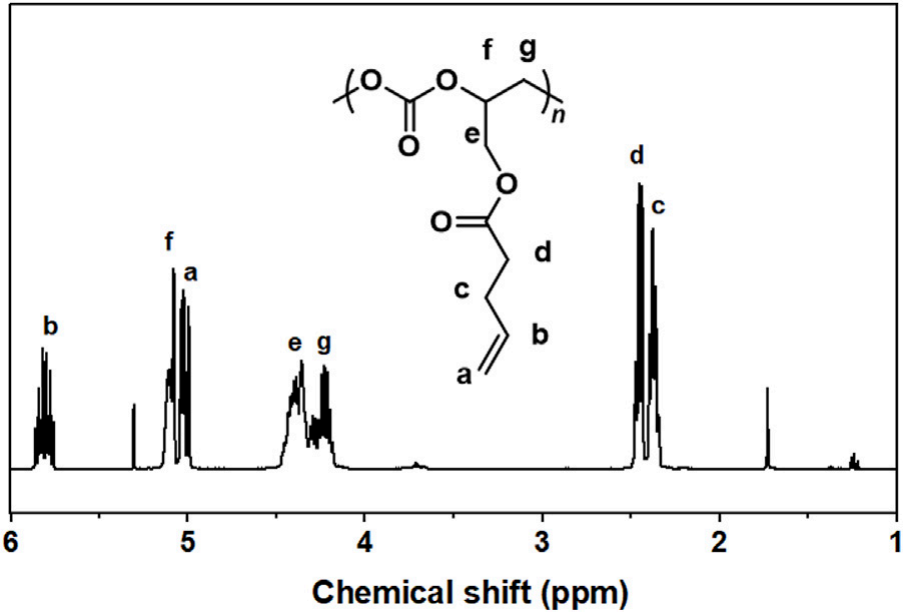
^1^H NMR spectra of pure products of SalenCoCl/PPNCl catalyzed polymerization (CDCl_3_, 400 MHz).

For crude products obtained from GPA/CO_2_ copolymerization catalyzed by SalenCoCl/PPNCl system, the peaks at 5.1 and 4.2 ppm were attributed to the protons on the methyl and methylene of the carbonate linkage, respectively, while the peaks of 4.9, 4.6 and 4.3 ppm were attributed to the protons on the methyl and methylene of the five-membered cyclic carbonate. The peaks at 3.5–3.8 ppm were attributed to the protons in the polyether chain segment (Figure 6). The ^1^H NMR spectrum of the purified product showed that the peaks of cyclic carbonate (j, k, l) at 4.9 ppm, 4.6 ppm, and 4.3 ppm completely disappeared, and the pure polycarbonate was obtained, as shown in Figure 7.

### 3.3 Catalytic analysis of GPA/CO_2_ copolymerization

As shown in Table 1, GPA/CO_2_ copolymerization catalyzed by SalenCoCl/PPNCl was conducted under various reaction times and temperatures. The conversion of the epoxides increased with extending reaction time (entries 1 and 2), reaching nearly 100% after 12 h. The polymer selectivity of the reaction was 92%, and almost no polyether linkage was formed. When the reaction time was extended to 24 h, the polymer selectivity decreased to 79% (entry 4), which was mainly due to the unstable structure of Salen (III)CoCl during the reaction. A portion of SalenCo(III) in the system was reduced to SalenCo(II) which might catalyze the cycloaddition reaction to form cyclic carbonate instead of the copolymerization to form polycarbonate, resulting in a decrease in polymer selectivity. In addition, the molecular weight of the polycarbonates declined with the prolongation of the reaction time, falling from 15.9 kg/mol at 12 h to 6.3 kg/mol at 48 h (entries 2–5). The decrease of the molecular weight was attributed to the back-biting reaction of the polycarbonate chain segment to form cyclic carbonate. Increasing the reaction temperature to 40°C (entry 8) decreased the polymer selectivity to 23% after 8 h of reaction, indicating that the stability of SalenCoCl declines as the temperature rises, leading to the generation of a large number of cyclic carbonate by-products.

To prevent side reactions, we lowered the reaction temperature to 0°C (entries 6, 7), and the polymer selectivity of the reaction increased to 95%. However, the catalytic activity of SalenCoCl/PPNCl was also reduced under 0°C, and the conversion of epoxide was only 76% after 48 h. Similarly, the molecular weight of the polymers also decreased, but it showed an increasing tendency with the prolongation of the reaction time. The molecular weights of polycarbonates gradually increased and reached 17.1 kg/mol at 5 MPa (entries 3, 9 and 10).

### 3.4 Terpolymerization of GPA, PO and CO_2_

**FIGURE 8:**
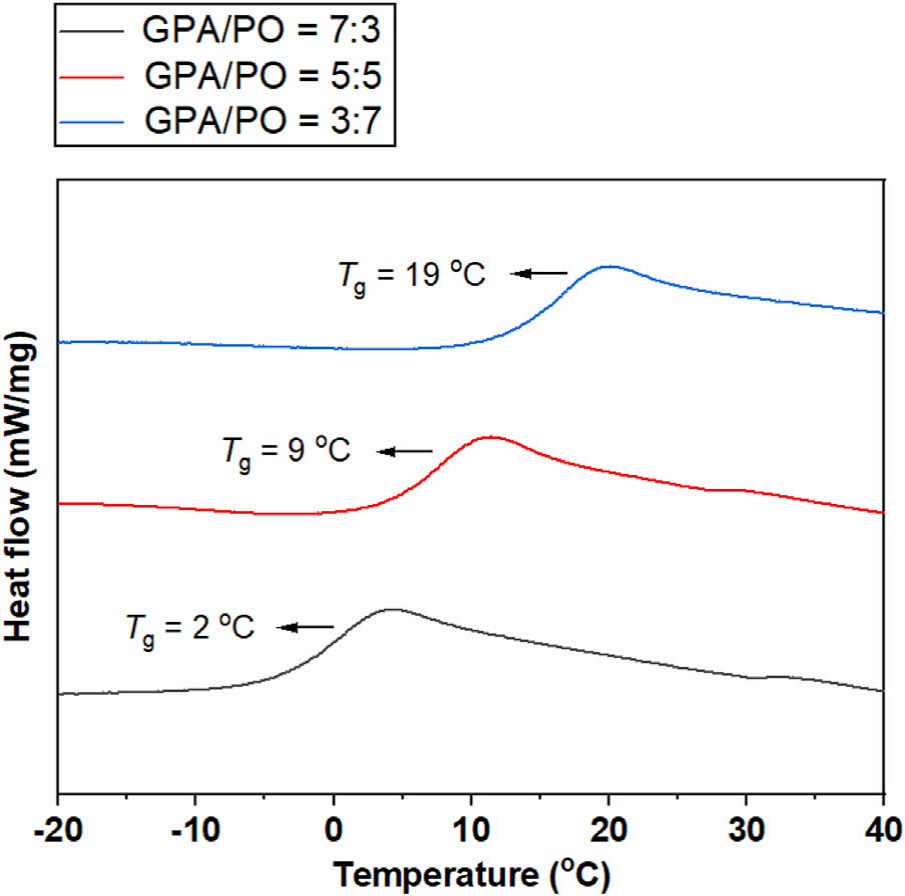
DSC curves of the polycarbonates obtained from the terpolymerization of GPA, PO, and CO_2_.

Thermal analysis of GPA/CO_2_ showed that the glass transition temperature (*T*_g_) of polycarbonate was −36°C, which was significantly lower than that of CO_2_/PO copolymer, most likely due to internal plasticization effects or micro-Brownian motion of the long alkyl side chains of GPA with ester groups (Okada et al., 2011). The terpolymerization of PO, GPA and CO_2_ was an effective method for achieving a balance between the hard and soft polymer segments. To adjust the *T*_g_ of CO_2_-based polycarbonate, a series of PO/GPA/CO_2_ terpolymer were synthesized via terpolymerization. When the mole ratio of GPA/PO changed from 7:3 to 3:7, the glass transition temperature (*T*_*g*_) of the polymers rose from 2°C to 19 °C (Figure 8).

### 3.5 Post-modification of polycarbonates

**FIGURE 9:**
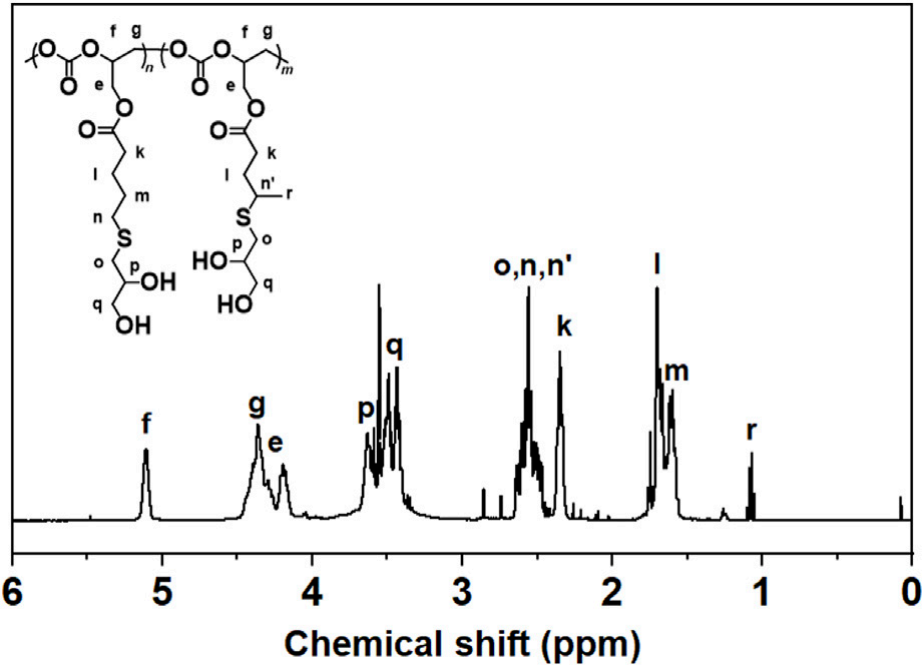
^1^H NMR spectra of the post-modification products (C_4_D_8_O, 400 MHz).

The vinyl groups in the side chain of the polymers offer the possibility of constructing functional polycarbonates through post-modification. 1-thioglycerol was successfully linked to the side chain of the polycarbonates via a radical-mediated thiol-ene click reaction, which was confirmed by ^1^H NMR spectrum (Figure 9). The peaks attributed to the vinyl groups at 5.2 ppm (a) and 5.8 ppm (b) completely disappeared along with appearance of new peaks which were attributed to 1-thioglycerol at 3.5 ppm (p, q), indicating complete conversion of the alkene groups via thiol-ene click reaction. In general, the thiol-ene click reaction occurs predominately with anti-Markovnikov regioselectivity. However, a hydrogen signal attributed to the methyl at 1.1 ppm (r) was found in the post-modification products, indicating the presence of partial Markovnikov regioselectivity in the thiol-ene click process.

**FIGURE 10:**
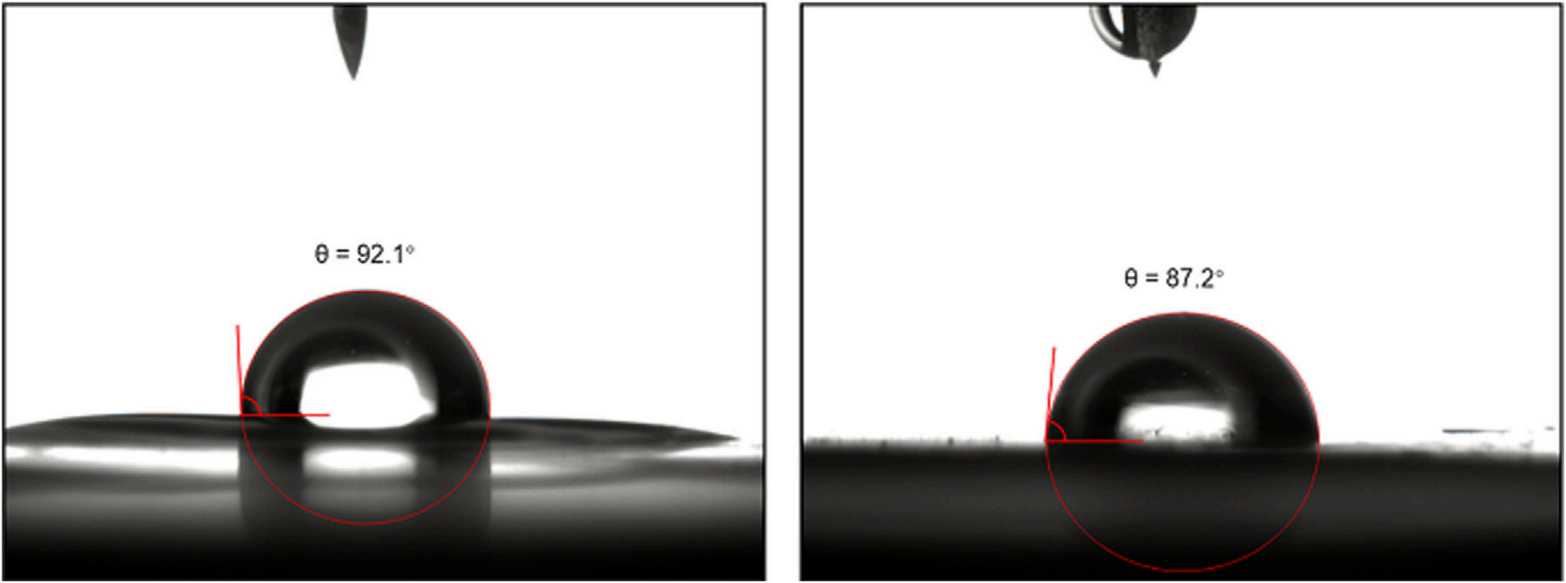
The contact angle measurements of GPA polycarbonates before (left) and after (right) the click reaction.

Contact angle measurements were conducted on GPA polycarbonates before and after the click reaction (Figure 10). It was observed that the contact angle of the unmodified polycarbonate increased from 92.1° to 87.2° after post-modification. This increase in contact angle indicates that the modified polymers became more hydrophilic compared to the unmodified polymers. The introduction of the hydroxyl group from 1-thioglycerol contributed to this increase in hydrophilicity. Therefore, the post-modification of polycarbonates with vinyl side groups has extensive potential applications in the preparation of biobased functional materials, such as antibacterial materials.

## 4 Conclusion

In this study, biobased 4-PA was employed to synthesize GPA, which was subsequently utilized in the synthesis of biobased polycarbonates through copolymerization with CO_2_. In addition, the effect of reaction conditions, such as reaction temperature, reaction time, and CO_2_ pressure, on the conversion of epoxides and the molecular weight of polycarbonates was thoroughly examined. Terpolymerization of GPA/PO/CO_2_ was also performed to regulate the glass transition temperature (*T*_g_) of the CO_2_-based polycarbonates in order to meet the needs of different applications. We also provide an example of successfully modifying the hydrophilicity of the polymer by reacting GPA polycarbonate with 1-thioglycerol via thiol-ene click chemistry. The introduction of hydroxyl groups in the side chains successfully reduces the contact angle from 92.1° to 87.2°. Biobased polycarbonates derived from CO_2_ and biobased 4-PA offer a new direction for preparing functional polycarbonates with reactive side chains, enabling post-modification for additional functionalities, such as antibacterial and antifouling properties.

## Data Availability

The original contributions presented in the study are included in the article/Supplementary Material, further inquiries can be directed to the corresponding author.

## References

[B1] Aida T. Inoue S. (1983). Activation of carbon dioxide with aluminum porphyrin and reaction with epoxide. Studies on (tetraphenylporphinato)aluminum alkoxide having a long oxyalkylene chain as the alkoxide group. J. Am. Chem. Soc. 105, 1304–1309. 10.1021/ja00343a038

[B2] Al-Naji M. Yepez A. Balu A. M. Romero A. A. Chen Z. Wilde N. (2016). Insights into the selective hydrogenation of levulinic acid to γ-valerolactone using supported mono- and bimetallic catalysts. J. Mol. Catal. A:Chem. 417, 145–152. 10.1016/j.molcata.2016.03.015

[B3] Al-Naji M. Puertolas B. Kumru B. Cruz D. Baumel M. Schmidt B. (2019). Sustainable continuous flow valorization of γ‐valerolactone with trioxane to α‐Methylene‐γ‐Valerolactone over basic beta zeolites. Chemsuschem 12, 2628–2636. 10.1002/cssc.201900418 30994965

[B4] Aresta M. Dibenedetto A. Angelini A. (2013). The changing paradigm in CO_2_ utilization. J. CO_2_ Util. 3-4, 65–73. 10.1016/j.jcou.2013.08.001

[B5] Artz J. Muller T. E. Thenert K. Kleinekorte J. Meys R. Sternberg A. (2018). Sustainable conversion of carbon dioxide: An integrated review of catalysis and life cycle assessment. Chem. Rev. 118, 434–504. 10.1021/acs.chemrev.7b00435 29220170

[B6] Byrne C. M. Allen S. D. Lobkovsky E. B. Coates G. W. (2004). Alternating copolymerization of limonene oxide and carbon dioxide. J. Am. Chem. Soc. 26, 11404–11405. 10.1021/ja0472580 15366863

[B7] Chang C. Qin Y. S. Luo X. L. Li Y. B. (2017). Synthesis and process optimization of soybean oil-based terminal epoxides for the production of new biodegradable polycarbonates via the intergration of CO_2_ . Ind. Crops Prod. 99, 34–40. 10.1016/j.indcrop.2017.01.032

[B8] Coates G. W. Moore D. R. (2004). Discrete metal-based catalysts for the copolymerization CO_2_ and epoxides: Discovery, reactivity, optimization, and mechanism. Angew. Chem. Int. Ed. 43, 6618–6639. 10.1002/anie.200460442 15558659

[B9] Cohen C. T. Chu T. Coates G. W. (2005). Cobalt catalysts for the alternating copolymerization of propylene oxide and carbon dioxide:combining high activity and selectivity. J. Am. Chem. Soc. 127, 10869–10878. 10.1021/ja051744l 16076192

[B10] Cui S. Q. Qin Y. S. Li Y. B. (2017). Sustainable approach for the synthesis of biopolycarbonates from carbon dioxide and soybean oil. Acs Sustain. Chem. Eng. 5, 9014–9022. 10.1021/acssuschemeng.7b01819

[B11] Darensbourg D. J. (2017). Making plastics from carbon dioxide: Salen metal complexes as catalysts for the production of polycarbonates from epoxides and CO_2_ . Chem. Rev. 107, 2388–2410. 10.1021/cr068363q 17447821

[B12] Gibson D. H. (1996). The organometallic chemistry of carbon dioxide. Chem. Rev. 96, 2063–2096. 10.1021/cr940212c 11848822

[B13] Han J. (2016). A bio-based ‘green’ process for catalytic adipic acid production from lignocellulosic biomass using cellulose and hemicellulose derived γ-valerolactone. Energy Convers. Manag. 129, 75–80. 10.1016/j.enconman.2016.10.019

[B14] Hauenstein O. Agarwal S. Greiner A. (2016). Bio-based polycarbonate as synthetic toolbox. Nat. Commun. 7, 11862. 10.1038/ncomms11862 27302694 PMC4912624

[B15] Hu Y. X. Qiao L. J. Qin Y. S. Zhao X. J. Chen X. S. Wang X. H. (2009). Synthesis and stabilization of novel aliphatic polycarbonate from renewable resource. Macromolecules 42, 9251–9254. 10.1021/MA901791A

[B16] Huang J. Worch J. C. Dove A. P. Coulembier O. (2020). Update and challenges in carbon dioxide-based polycarbonate synthesis. ChemSusChem 13, 469–487. 10.1002/cssc.201902719 31769174

[B17] Iglesias J. Martínez-Salazar I. Maireles-Torres P. Martin Alonso D. Mariscal R. López Granados M. (2020). Advances in catalytic routes for the production of carboxylic acids from biomass: A step forward for sustainable polymers. Chem. Soc. Rev. 49, 5704–5771. 10.1039/D0CS00177E 32658221

[B18] Isikgor F. H. Becer C. R. (2015). Lignocellulosic biomass: A sustainable platform for the production of bio-based chemicals and polymers. Polym. Chem. 6, 4497–4559. 10.1039/C5PY00263J

[B19] Jing Y. X. Guo Y. Xia Q. N. Liu X. H. Wang Q. Y. (2019). Catalytic production of value-added chemicals and liquid fuels from lignocellulosic biomass. Chem 5, 2520–2546. 10.1016/j.chempr.2019.05.022

[B20] Jung J. H. Ree M. Chang T. (1999). Copolymerization of carbon dioxide and propylene oxide using an aluminum porphyrin system and its components. J. Polym. Sci. A Polym. Chem. 37, 3329–3336. 10.1002/(SICI)1099-0518(19990815)37:16<3329::AID-POLA31>3.0.CO;2-Q

[B21] Klaus S. Lehenmeier M. W. Anderson C. E. Rieger B. (2010). Recent advances in CO_2_/epoxide copolymerization—new strategies and cooperative mechanisms. Coord. Chem. Rev. 255, 1460–1479. 10.1016/j.ccr.2010.12.002

[B22] Kozak C. M. Ambrose K. Anderson T. S. (2018). Copolymerization of carbon dioxide and epoxides by metal coordination complexes. Coord. Chem. Rev. 376, 565–587. 10.1016/j.ccr.2018.08.019

[B23] Lin E. Y. Lu C. (2021). Development perspectives of promising lignocellulose feedstocks for production of advanced generation biofuels: A review. Renew. Sustain. Energy Rev. 136, 110445. 10.1016/j.rser.2020.110445

[B24] Lu X. B. Darensbourg D. J. (2012). Cobalt catalysts for the coupling of CO_2_ and epoxides to provide polycarbonates and cyclic carbonates. Chem. Soc. Rev. 41, 1462–1484. 10.1039/c1cs15142h 21858339

[B25] Lu X. B. Wang Y. (2004). Highly active, binary catalyst systems for the alternating copolymerization of CO_2_ and epoxides under mild conditions. Angew. Chem. Int. Ed. 43, 3574–3577. 10.1002/anie.200453998 15293249

[B26] Lu X. B. Shi L. Wang Y. M. Zhang R. Zhang Y. J. Peng X. J. (2006). Design of highly active binary catalyst systems for CO_2_/epoxide copolymerization: Polymer selectivity, enantioselectivity, and stereochemistry control. J. Am. Chem. Soc. 128, 1664–1674. 10.1021/ja056383o 16448140

[B27] Nobbs J. D. Zainal N. Z. B. Tan J. Drent E. Stubbs L. P. LiLim C. S. C. Y. (2016). Bio-based pentenoic acids as intermediates to higher value-added mono- and dicarboxylic acids. ChemistrySelect 1, 539–544. 10.1002/slct.201600136

[B28] Okada A. Kikuchi S. Yamada T. (2011). Alternating copolymerization of propylene oxide/alkylene oxide and carbon dioxide: Tuning thermal properties of polycarbonates. Chem. Lett. 40, 209–211. 10.1246/cl.2011.209

[B29] Omae I. (2012). Recent developments in carbon dioxide utilization for the production of organic chemicals. Coord. Chem. Rev. 256, 1384–1405. 10.1016/j.ccr.2012.03.017

[B30] Parrino F. Fidalgo A. Palmisano L. Ilharco L. M. Pagliaro M. Ciriminna R. (2018). Polymers of limonene oxide and carbon dioxide: Polycarbonates of the solar economy. Acs Omega 3, 4884–4890. 10.1021/acsomega.8b00644 31458704 PMC6641970

[B31] Plajer A. J. Williams C. K. (2022). Heterocycle/heteroallene ring-opening copolymerization: Selective catalysis delivering alternating copolymers. Angew. Chem. Int. Ed. Engl. 61, e202104495. 10.1002/anie.202104495 34015162 PMC9298364

[B32] Poland S. J. Darensbourg D. J. (2017). A quest for polycarbonates provided via sustainable epoxide/CO_2_ copolymerization processes. Green Chem. 19, 4990–5011. 10.1039/C7GC02560B

[B33] Qin Y. S. Wang X. H. (2010). Carbon dioxide-based copolymers: Environmental benefits of PPC, an industrially viable catalyst. Biotechnol. J. 5, 1164–1180. 10.1002/biot.201000134 21058318

[B34] Raj T. Chandrasekhar K. Banu R. Yoon J. J. Kumar G. Kim S. H. (2021). Synthesis of γ-valerolactone (GVL) and their applications for lignocellulosic deconstruction for sustainable green biorefineries. Fuel 303, 121333. 10.1016/j.fuel.2021.121333

[B35] Sakakura T. Choi J. C. Yasuda H. (2007). Transformation of carbon dioxide. Chem. Rev. 107, 2365–2387. 10.1021/cr068357u 17564481

[B36] Somerville C. Youngs H. Taylor C. Davis S. C. Long S. P. (2010). Feedstocks for lignocellulosic biofuels. Science 329, 790–792. 10.1126/science.1189268 20705851

[B37] Wang Y. Y. Fan J. W. Darensbourg D. J. (2015). Construction of versatile and functional nanostructures derived from CO_2_‐based polycarbonates. Angew. Chem. Int. Ed. 54, 10206–10210. 10.1002/anie.201505076 26177634

[B38] Wang Y. Zhao Y. J. Ye Y. S. Peng H. Y. Zhou X. P. Xie X. L. (2018). A one-step route to CO_2_-based block copolymers by simultaneous ROCOP of CO_2_/epoxides and RAFT polymerization of vinyl monomers. Angew. Chem. Int. Ed. 57, 3593–3597. 10.1002/anie.201710734 29392807

[B39] Wang F. Q. Ouyang D. H. Zhou Z. Y. Page S. J. Liu D. H. Zhao X. B. (2021). Lignocellulosic biomass as sustainable feedstock and materials for power generation and energy storage. J. Energy Chem. 57, 247–280. 10.1016/j.jechem.2020.08.060

[B40] Xian W. Q. Yuan J. Xie Z. B. Ou W. Liu X. X. Liu B. H. (2021). Synthesis and properties of CO_2_ copolymer-based waterborne polyurethane with high solid content. J. Polym. Res. 28, 254. 10.1007/s10965-021-02616-9

[B41] Yaashikaa P. R. Kumar P. S. Varjani S. J. Saravanan A. (2019). A review on photochemical, biochemical and electrochemical transformation of CO_2_ into value-added products. J. CO_2_ Util. 33, 131–147. 10.1016/j.jcou.2019.05.017

[B42] Yang J. Dong J. C. Wang Y. P. Zhang X. Liu B. Y. Shi H. (2021). Phase transition and crystallization of bio-based comb-like polymers based on renewable castor oil-derived epoxides and CO_2_ . Macromolecules 54, 8503–8511. 10.1021/acs.macromol.1c01362

[B43] Zhang Y. Y. Zhang X. H. Wei R. J. Du B. Y. Fan Z. Q. Qi Q. R. (2014). Synthesis of fully alternating polycarbonate with low *T* _g_ from carbon dioxide and bio-based fatty acid. Rsc. Adv. 4, 36183–36188. 10.1039/C4RA06157H

